# MRI of Whole Rat Brain Perivascular Network Reveals Role for Ventricles in Brain Waste Clearance

**DOI:** 10.1038/s41598-019-44938-1

**Published:** 2019-08-07

**Authors:** Kulam Najmudeen Magdoom, Alec Brown, Julian Rey, Thomas H. Mareci, Michael A. King, Malisa Sarntinoranont

**Affiliations:** 1 Department of Mechanical and Aerospace Engineering, University of Florida, Gainesville, FL USA; 2 Department of Biochemistry and Molecular Biology, University of Florida, Gainesville, FL USA; 3 Department of Pharmacology and Therapeutics, University of Florida, Gainesville, FL USA; 4 Department of Veterans Affairs Medical Center, Gainesville, FL USA

**Keywords:** Magnetic resonance imaging, Brain

## Abstract

Investigating the mechanisms by which metabolic wastes are cleared from nervous tissue is important for understanding natural function and the pathophysiology of several neurological disorders including Alzheimer’s disease. Recent evidence suggests clearance may be the function of annular spaces around cerebral blood vessels, called perivascular spaces (PVS), through which cerebrospinal fluid (CSF) is transported from the subarachnoid space into brain parenchyma to exchange with interstitial fluid (also known as the glymphatic system). In this work, an MRI-based methodology was developed to reconstruct the PVS network in whole rat brain to better elucidate both PVS uptake and clearance pathways. MR visible tracer (Gd-albumin) was infused *in vivo* into the CSF-filled lateral ventricle followed by *ex vivo* high-resolution MR imaging at 17.6 T with an image voxel volume two orders of magnitude smaller than previously reported. Imaged tracer distribution patterns were reconstructed to obtain a more complete brain PVS network. Several PVS connections were repeatedly highlighted across different animals, and new PVS connections between ventricles and different parts of the brain parenchyma were revealed suggesting a possible role for the ventricles as a source or sink for solutes in the brain. In the future, this methodology may be applied to understand changes in the PVS network with disease.

## Introduction

Transport of nutrients and removal of waste from the brain interstitial space is essential to maintain homeostasis in the nervous system. Since the brain has no lymphatic vessels, alternative mechanisms for clearance propose that waste transport is a natural function of perivascular spaces (PVS), which are annular fluid channels surrounding blood vessels^1,2^. Malfunctioning of PVS clearance pathways has been implicated in the pathophysiology of Alzheimer’s disease^3^, type 2 diabetes^4^, and traumatic brain injury^5^.

Nedergaard and co-workers have conducted *in vivo* two photon imaging of the brain surface following injection of fluorescent tracers into mouse cisterna magna^1^. Based on their observations, they propose a glymphatic theory of perivascular transport in which CSF enters the brain parenchyma *via* perivascular spaces surrounding cerebral artery/arterioles. This fluid is then thought to be driven through interstitial spaces where it clears waste products as it exits back into CSF through perivenous spaces. Ultimately, waste cargo is drained into arachnoid granulations which exchange with venous sinuses and/or into cervical lymph nodes along the olfactory nerve sheath^6^, or through lymphatic vessels found in the meninges^7^. This direct connection between CSF and brain parenchyma also may open new avenues for drug delivery to the brain bypassing the blood-brain barrier via intrathecal drug infusion^8,9^ into the CSF-filled spaces.

Previous perivascular transport studies have focused mainly on external subregions of the brain, such as the cortex, due to the limited penetration depth of the optical techniques used. Uptake of tracers has been observed along surface cortical blood vessels^1,10^. However, clearance routes for these tracers have not been measured as readily in part due to a lack of high-resolution imaging techniques to study perivascular transport deep within brain parenchyma and along exit routes out of the brain. Research in this area is actively ongoing and some studies present conflicting findings. Electron microscopy studies conducted in human brain samples have found that veins in the cerebral cortex and basal ganglia appear to lack perivascular spaces, confounding understanding of clearance pathways central to the glymphatic theory^11^. Also, the convective current of CSF-interstitial fluid (ISF) mixture proposed in the glymphatic theory is challenged by several computational transport modeling and experimental studies which argue diffusion or dispersion might be sufficient to explain the observed tracer patterns^12–14^.

Imaging methods that allow complete mapping of larger sections of the perivascular network in the brain will be important in understanding connectivity and transport along these channels, in normal function, and in pathological states. Given the limited coverage of current CSF mediated drug delivery to the brain, mapping the whole brain PVS network may also help develop new drug delivery strategies by identifying major transport routes. Magnetic resonance imaging (MRI) is well suited for the purpose of whole brain imaging, but only a few MRI studies address PVS pathways in the whole brain. Previous mapping studies have used MR contrast agents injected into CSF spaces in rats *in vivo*^9,15^ and humans^16^. However, the spatial resolution of scans was not sufficient to capture PVS deep in the brain parenchyma which are estimated to range from 1–10 μm in fixed rat brains^17,18^.

This study introduces a method for imaging the whole rat brain perivascular network at high spatial resolution with the goal of providing improved visualization of the PVS network along smaller vessels in the brain interior. MR visible tracer was infused into the lateral ventricle *in vivo* followed by high resolution MR imaging *ex vivo* with an MR image voxel volume two orders of magnitude smaller than previously reported^9,15^ allowing for visualization of new PVS connections between brain parenchyma and ventricles.

## Results

**Figure 1:**
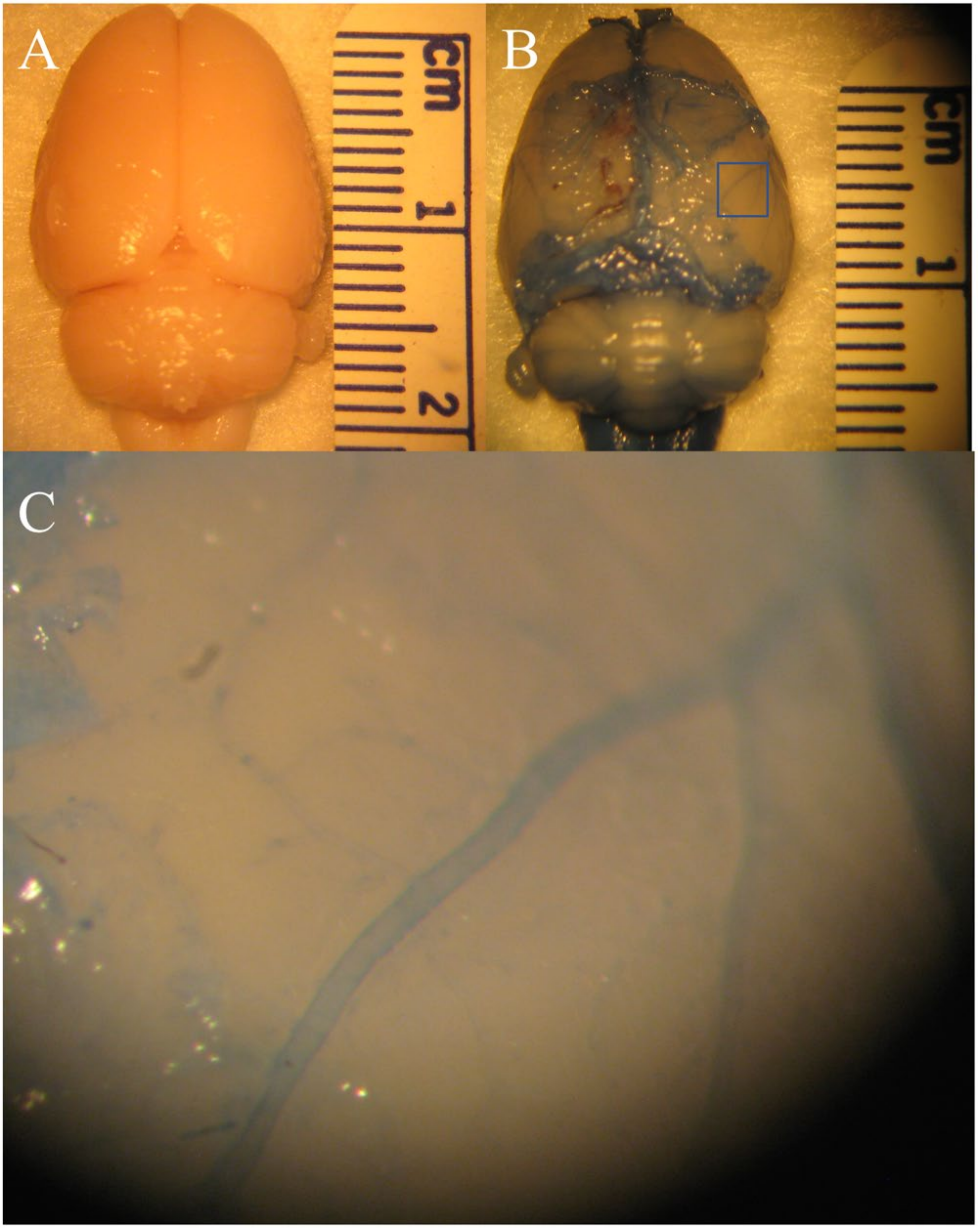
Perivascular uptake of the tracer visualized in brightfield images obtained using a dissection microscope eyepiece camera. (**A**) Naïve rat brain, (**B**) Rat brain with Gd-Alb-EB infused into the lateral ventricle with attached dura. (**C**) Magnified view of the blue rectangular region of interest drawn in (**B**) showing perivascular tracer labeling along cortical blood vessels of a tracer-infused rat.

Perivascular tracer uptake was visually confirmed with brightfield optical images of the brain acquired using a dissection microscope eyepiece camera. The top row of Fig. 1 shows low magnification whole brain images of a representative naïve control and a tracer-infused rat brain, while the bottom row shows the region around a pial blood vessel in the cortex of the tracer infused brain obtained with a higher power objective. The blue outline along blood vessels corresponds to perivascular labeling by Evans blue dye bound to the Gd-albumin tracer.

**Figure 2:**
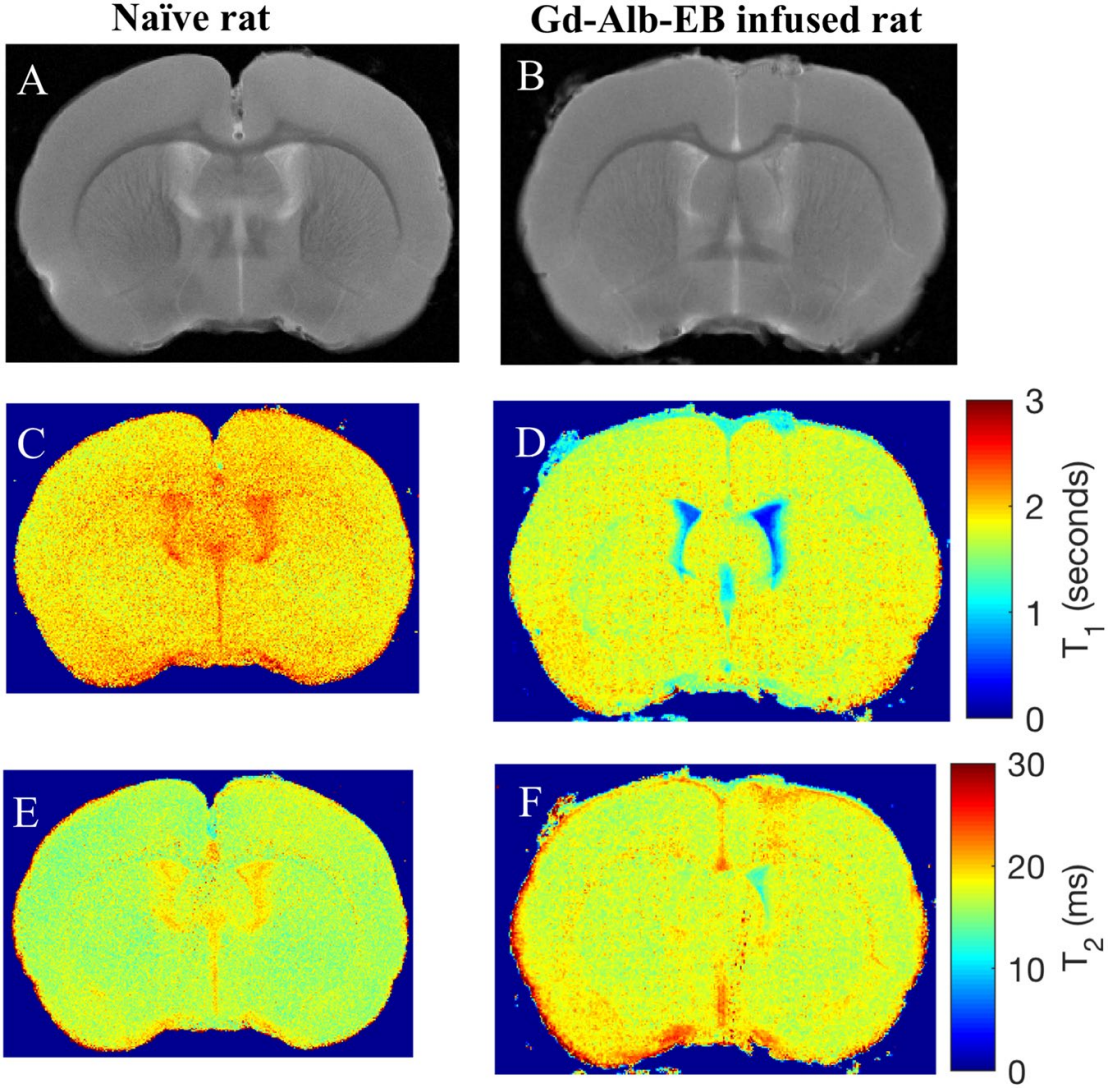
Tracer infusion into the lateral ventricle evidenced by reductions in T_1_ and T_2_ compared to naïve rats. (**A**,**B**) Proton density weighted images (TR = 5000 ms, TE = 9 ms), (**C**,**D**) quantitative T_1_, and (**E**,**F**) quantitative T_2_ maps of a slice from naïve rat brain and rat brain with Gd-Alb-EB infused into the lateral ventricle.

Successful tracer infusion and absence of pre-existing abnormalities in the brain was verified using quantitative T_1_ and T_2_ maps, respectively, acquired prior to high resolution PVS imaging. Representative results from a naïve control and a tracer-infused rat are shown in Fig. 2, along with proton density images for anatomical reference. Images show reductions in T_1_ and T_2_ relaxation times in the lateral and third ventricle in the tracer-infused rat compared to the naïve rat. No backflow of the tracer was observed during infusion however tracer seeding along the needle track while inserting and removing the infusion needle was visible in the proton density image and T_1_ map.

**Figure 3:**
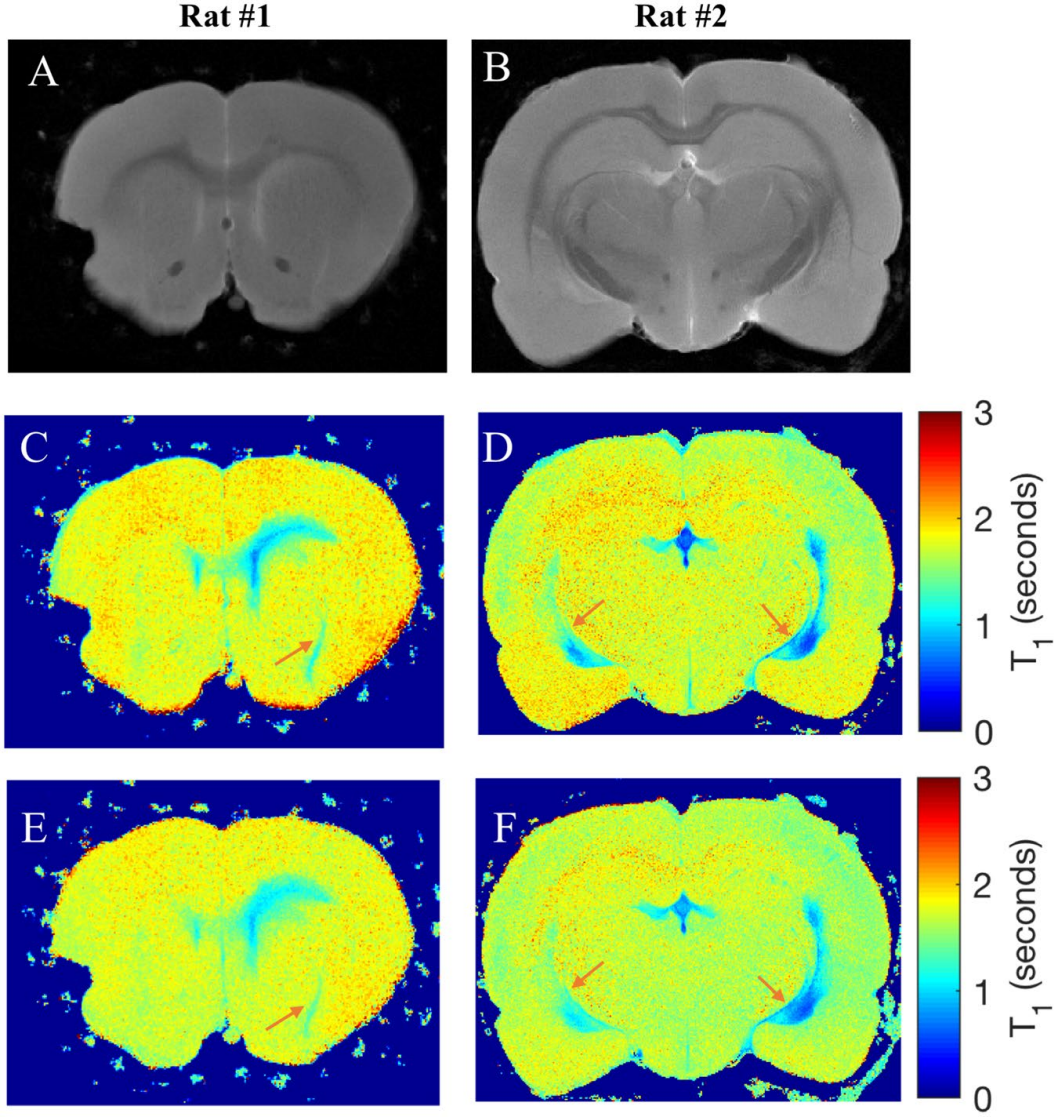
Preservation of perivascular labelling by the tracer in a 24 hr time period. (**A**,**B**) Proton density weighted images (TR = 5000 ms, TE = 9 ms) of a slice from two rats with Gd-Alb-EB infused into the lateral ventricle. Quantitative T_1_ maps acquired before (**C**,**D**) and after (**E**,**F**) high resolution 3D imaging (approximately 24 hours apart) to observe tracer diffusion. Orange arrows show T_1_ reductions in perivascular spaces persist after the 24-hour imaging period.

Preservation of perivascular tracer labelling with perfusion fixation was shown by comparing the quantitative T_1_ maps of two tracer-infused rats acquired 24 hours apart before and after 3D PVS imaging in Fig. 3. T_1_ reductions along PVS of major blood vessels were maintained over the 24-hour time period. The T_1_ map also showed diffuse spread of the tracer along corpus callosum close to the lateral ventricle. To quantify the effect of tracer diffusion, the infused lateral ventricle was segmented based on the region-grow algorithm to calculate the mean T_1_ inside the region for naïve and tracer-infused rats before and after PVS imaging. Proton density-weighted images were used to segment ventricles in the naïve rat since these provided better contrast than the T_1_ maps.

Mean T_1_ for the lateral ventricle in naïve control (n = 2) and tracer-infused rats before (n = 5) and after (n = 5) PVS imaging were 2159.58 ± 11.47, 662.51 ± 89.04 and 774.44 ± 89.89 ms with *p*-values equal to 2.4 × 10^−3^, 7.64 × 10^−5^ and 4.28 × 10^−5^, respectively. Mean T_1_ at the end of PVS imaging was approximately 17% higher than that before imaging with a *p*-value equal to 1.69 × 10^−4^.

**Figure 4:**
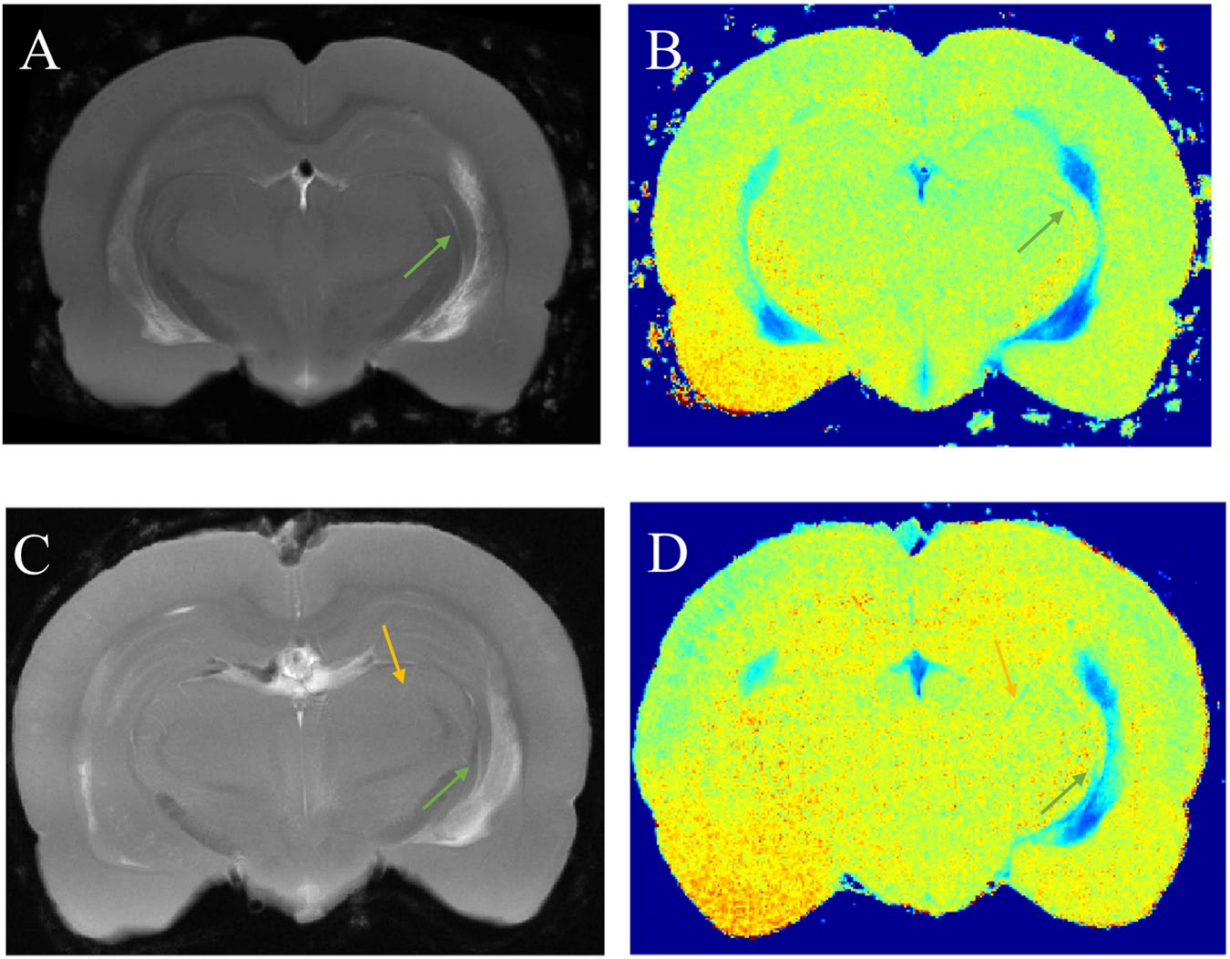
Co-localization of hyperintense perivascular spaces delineated in T_1_ weighted images with reductions in T_1_. (**A**,**C**) High resolution T_1_ weighted images from two rats used to reconstruct the perivascular network decimated in the slice direction by averaging to match the slice thickness of corresponding low resolution T_1_ map which is shown in (**B**,**D**). Perivascular spaces surrounding internal carotid artery branch (green arrows) and penetrating thalamic blood vessel (yellow arrows) in T_1_ weighted images and quantitative T_1_ maps are marked for both the rats.

Hyperintense regions in whole-brain scans were correlated with hypointense regions in 2D quantitative T_1_ scans to verify that the observed PVS signal enhancement in whole-brain images was due to gadolinium. High-resolution 3D PVS images of two tracer-infused rats were averaged in the slice dimension to match the slice thickness of 2D quantitative T_1_ scans for comparison. PVS along the internal carotid artery branch and a thalamic blood vessel were visible on both 3D scan and T_1_ maps as shown in Fig. 4.

**Figure 5:**
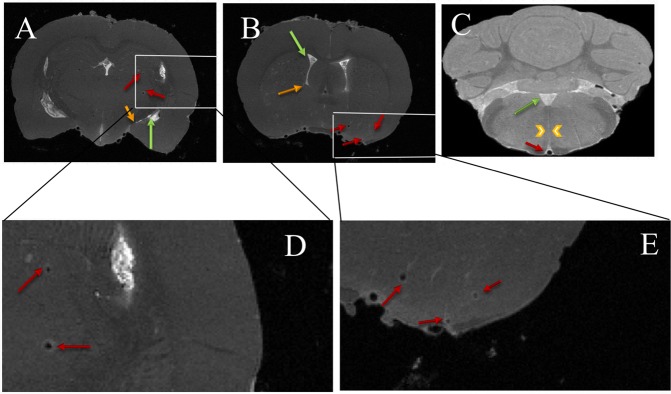
Perivascular tracer labeling and PVS connections between ventricles and brain parenchyma in individual image slices from the 3D image stack. (**A**) Ring enhancement around penetrating thalamic blood vessels (red arrows) showing the presence of tracer in PVS. Also shown are PVS around anterior choroidal artery (yellow arrow) connecting the lateral ventricle (green arrow) to the ventral brain surface. (**B**) Ring enhancement around vessels in cortex (red arrows) and PVS connections between lateral ventricle (green arrow) and caudate/putamen (yellow arrow). (**C**) Ring enhancement around the basilar artery (red arrow) and PVS around a paramedian branch of the basilar artery (yellow arrow head) connecting to the fourth ventricle (green arrow). (**D**,**E**) Enlarged sections of (**A**,**B**) outlined in white showing ring enhancement around blood vessels.

**Figure 6:**
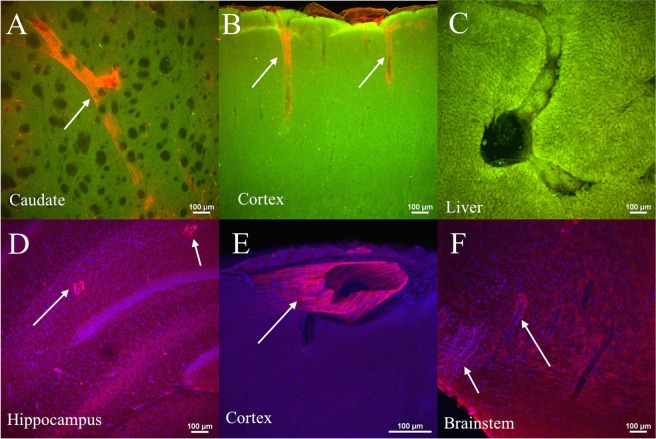
Laser confocal fluorescence images of different regions of the brain, and liver obtained after tracer infusion into the lateral ventricle and *ex vivo* MR imaging. (**A**–**C**) Tracer fluorescence images (red) overlaid with tissue autofluorescence image (green). (**D**–**F**) Tracer fluorescence images (red) overlaid with DAPI image (blue) for anatomical reference. Notice perivascular labelling (white arrows) shown by intense orange and pink regions in top and bottom row images respectively, and the absence of labeling in a hepatic blood vessel showing the tracer is abluminal in the PVS.

Localization of the tracer to PVS and exclusion from inside the blood vessel was verified by examining individual MRI slices from the high-resolution 3D scan, as shown in Fig. 5. The figure shows ring enhancement around blood vessels in different parts of the brain. Since this enhancement could also result from tracer binding to the luminal surface of blood vessels, possibly from tracer entering the bloodstream by natural CSF absorption in the venous sinuses, confocal imaging was performed on a hepatic blood vessel of a tracer infused rat. Tracer did not bind to the hepatic vessel wall from within the lumen as shown in Fig. 6C.

**Figure 7:**
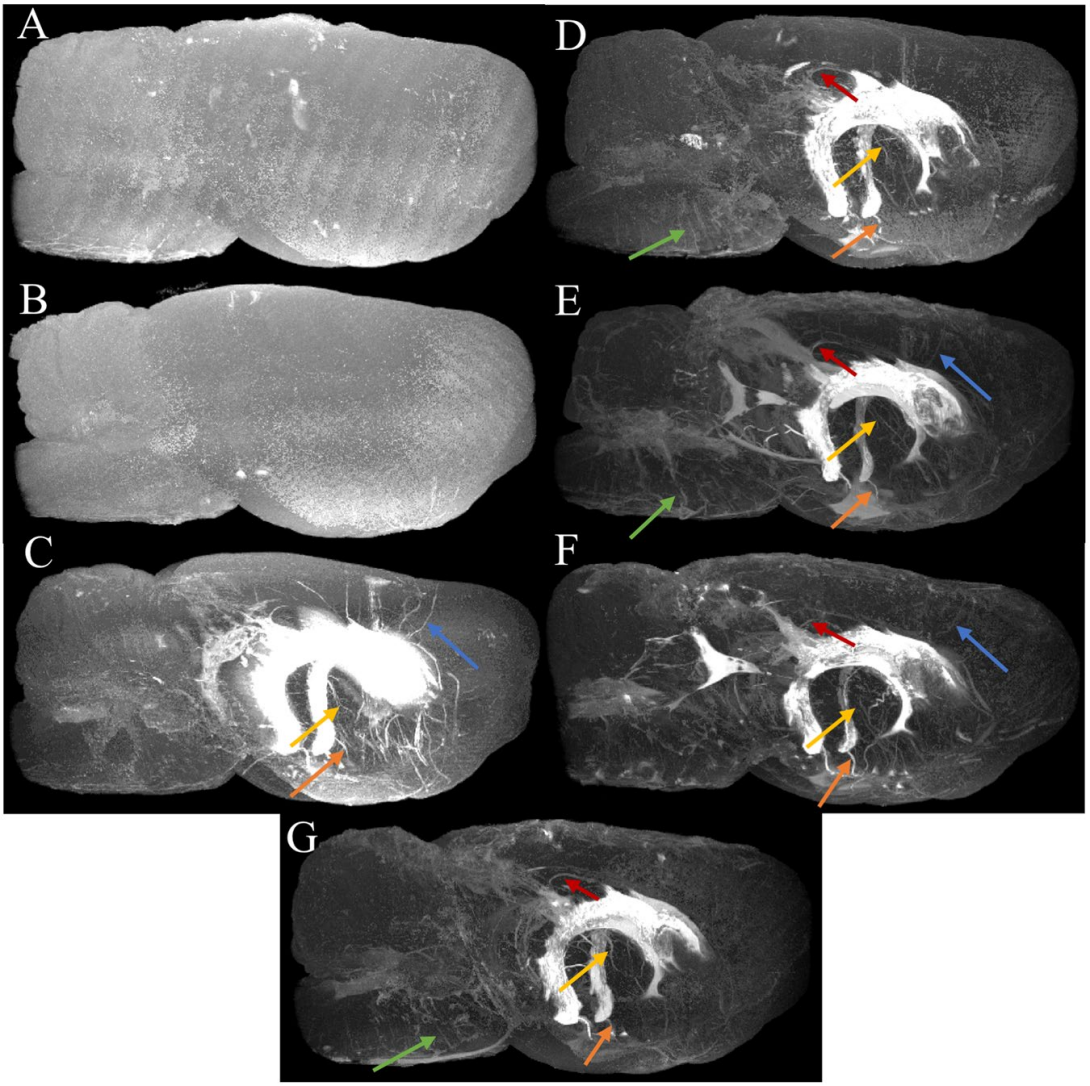
2D maximum intensity projection of the reconstructed 3D perivascular network from five rats registered to the template rat brain atlas. (**A**,**B**) Results of the analysis applied to the naïve rats and (**C**–**G**) to the tracer infused rats. Vessels with perivascular spaces that appear to be common in different rats are highlighted using colored arrows. PVS connecting lateral ventricles to the dorsal brain surface (red and blue arrow), ventral brain surface (orange arrow), deep brain structures (yellow arrow) are highlighted along with PVS in the brainstem (green arrow). 3D animations are provided in the supplemental data.

Confocal images in Fig. 6 also confirm the presence of tracer in the PVS throughout the brain including caudate, cerebral cortex, hippocampus and brainstem deep within the brain parenchyma. Two-dimensional projections of the whole-brain PVS network reconstructions for all 5 tracer-infused rats are shown in Fig. 7, which also includes the reconstruction performed on naïve rats to show any artifacts that might have resulted from the image analysis. Three-dimensional animation of representative naïve and PVS network is provided in the Supplementary Movies S1 and S2, respectively. The brain surface was captured in naïve rat brains without any vessel or ventricular outlining inside the parenchyma. A few hyperintense regions were observed due to susceptibility artifacts resulting from bubbles trapped within the fissures in the brain while preparing the sample for MR imaging.

Within tracer-infused brains, tracer was visible in the ventricles and hippocampal/cerebellar fissures. There was a higher density of PVS observed within the ventral brain surface (bottom) compared to the dorsal side (top) as shown in Fig. 7. Increased signal enhancement in the dorsal surface observed in a few rats may be due to tracer present in the attached dura. For reasons that are not yet fully understood, signal enhancement was absent in the cerebral aqueduct and fourth ventricle in three out of five rats. This might be due to accidental escape of tracer from the posterior ventricles during brain dissection or while placing the brain in the tube for MR imaging.

PVS pathways were found to connect different parts of the brain to the ventricles. PVS routes connecting the dorsal surface (shown by red and blue arrows), ventral surface (shown by orange arrows) and deep brain structures (shown by yellow arrows) to the lateral ventricle were reliably detected across rats. A connection between the lateral ventricle and ventral brain surface through PVS surrounding the anterior choroidal artery is shown in individual slice images from the 3D acquisition in Fig. 5A. PVS connections between branches of the basilar artery penetrating deep into the brainstem and fourth ventricle were also observed (Fig. 5D).

## Discussion

The objective of this study was to develop a methodology to map the PVS network in large regions of the rat brain at a high spatial resolution to better understand the connectivity of perivascular channels. The developed MRI approach is capable of capturing PVS along blood vessels deep into the brain parenchyma. New PVS routes between ventricles and brain parenchyma were found.

### MRI captures PVS routes

In 3D MRI scans, hyperintense regions along blood vessels were co-localized with hypointense regions in the T_1_ map to relate signal origin to the effect of contrast agent. PVS was resolved from the blood vessel lumen in individual 2D slices from the 3D stack of MR images indicating the tracer was outside the blood vessel and not inside. It is unlikely that detectable levels of tracer remained inside the blood vessel lumen since MR imaging was performed after vascular perfusion fixation. The selective presence of tracer outside the vessel lumen in the PVS could be confirmed in future studies through electron microscopy involving electron dense tracers such as colloidal gold^19^.

Characteristics of the perivascular network obtained in this study agree with what has been previously reported about these pathways. We observed a high density of PVS along blood vessels within the ventral surface of the brain. This pattern has been previously reported with intracisternal tracer infusion in rats and mice^9,10,15^. It is not entirely clear why there would be greater PVS density in this region of the brain. Possible explanations include smaller perivascular tracer influx at the dorsal surface due to CSF absorption by arachnoid granulations along the dorsal surface, the presence of larger caliber blood vessels within the ventral side with greater pulsatility^10^, directionality imposed by the displacement of CSF in the lateral ventricle and/or presence of more PVS in the ventral surface than the dorsal surface.

Diffusional transport does not explain tracer uptake patterns along the PVS. The possibility of axial tracer diffusion accounting for the observed connection between the ventricle and PVS was explored. Diffusion displacement of tracer in a cylindrical annulus (i.e. PVS) in the brain during the 40-minute infusion period is approximately 0.5 mm (D ~ 1.63 × 10^−11^ m^2^/s for albumin^20^) which is much smaller than the largest distance between the ventricles and brain surface (~6 mm) for which contrast enhancement was observed.

### PVS solute clearance via ventricles

**Figure 8:**
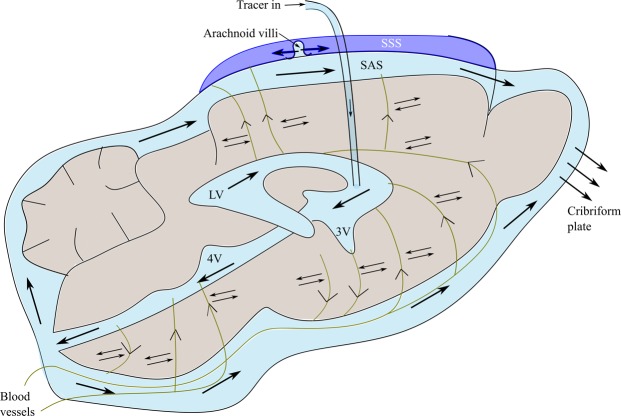
Proposed path for the transport of Gd-albumin tracer (black arrows) infused into the lateral ventricle. Tracer infused into the lateral ventricle reaches the subarachnoid space (SAS) either through the PVS in the parenchyma around it or through the CSF macro circulation in the ventricular system. A portion of the tracer in the SAS drains into the cribriform plate and superior sagittal sinus (SSS), while the rest is transported back into the brain parenchyma through PVS surrounding the blood vessels (green lines) in the brain surface. The PVS surrounding these blood vessels eventually join the macro CSF circulation in the ventricular system. Solute removal from the parenchyma could be facilitated by constant exchange between ISF in the parenchyma and CSF in the PVS by diffusion. Tracer also diffuses from the ventricles and SAS into the adjacent parenchyma (not shown). Arrows along PVS indicate possible directions of transport into and out of the brain. Abbreviations: LV - lateral ventricle, 3 V - third ventricle and 4 V - fourth ventricle.

The high resolution PVS network obtained in this study also emphasizes the important role ventricles may play in PVS mediated solute clearance from brain parenchyma. Given the current debate on the existence of flow in the parenchyma outside the PVS, the ventricles could be viewed as PVS-accessible routes for the rapid clearance of solutes in the parenchyma. Newly found PVS pathways connecting directly to the ventricles from blood vessels penetrating the dorsal and ventral surface of the brain suggest that the PVS may eventually join with CSF macrocirculation in the ventricles and subarachnoid space while constantly exchanging with the ISF by diffusion, thus providing a rapid route for solutes in the PVS and parenchyma (Fig. 8). This is a more direct route than solute transport along perivenous spaces (subarachnoid space – PVS – ventricles or vice-versa).

Since the focus of our study was to map out PVS pathways and show connectivity within deep brain structure, directionality of transport was not determined. Tracer may have entered the PVS from either the ventricles and/or the subarachnoid space after traversing through the macrocirculation.

A role for cerebral ventricles in brain waste clearance has been recently considered by Pizzo *et al*.^9^. Using *ex vivo* confocal imaging, fluorescent tracers were found in lateral ventricles following cistern magna infusion. It was hypothesized that this accumulation resulted from PVS transport associated with blood vessels feeding the choroid plexus, given the CSF flow at the cisterna magna is away from the lateral ventricles. The ventricular system has been known for several decades to function as a clearance route for brain metabolites. Studies involving direct CSF sampling from lateral ventricles in cats and monkeys have shown that serotonin and histamine are transported from the brain into CSF for clearance^21,22^.

### Tissue fixation

Adequate fixation resulting in sufficient cross linking between the albumin tracer and extracellular matrix is important for maintaining the perivascular labelling post infusion. However, the MR image contrast between CSF and brain tissue was significantly altered due to T_1_ reductions observed with formaldehyde-based fixation^23^. This was clearly visible in the T_1_ maps of naïve rats, where tissue and CSF had very similar T_1_ values. Reduction in the contrast due to fixation was partly compensated using a high dose of the contrast agent, and an ultra-high strength magnet. It has been reported that the T_1_ of brain tissue increases logarithmically with field strengths^24^. Since the MR signal enhancement in a medium due to the contrast agent is directly proportional to the reduction in the T_1_ relaxation rate relative to the pre-contrast values, an increase in baseline T_1_ would be expected to increase the signal enhancement and improve sensitivity. This could however be partly offset by reductions in longitudinal relaxivity (r_1_) of the contrast agent with field strength^25^, and dephasing due to transverse relaxivity (r_2_) which was minimized by shortening the echo time. A combination of these factors is reflected by almost 70% and 26% reductions in T_1_ and T_2_ respectively at the infusion site. T_1_ values did increase slightly at the infusion site at the end of 24-hour high resolution PVS imaging perhaps due to tracer diffusion from the lateral ventricles into the adjacent parenchyma. Lack of anchoring proteins in the lateral ventricles could have promoted diffusion, however the tracer in the PVS should be fairly intact from the fixation process.

### Caveats & future work

Inherently low sensitivity of MRI limits the ability to fully resolve PVS which is in many places smaller in scale than the voxel size obtained in this study. Partial volume effects could be minimized by increasing the MRI scan resolution further using even higher field strength magnets. Advances in MR hardware such as phased array RF coils may boost the signal-to-noise ratio and/or microcoils may be used to improve sensitivity to image brain sections larger than that allowed by optical techniques^26^. It should be noted that optical clearing techniques such as CLARITY^27^ and ScaleS^28^ exist which render the tissue transparent thus allowing very high resolution whole brain optical imaging. However, reference to surrounding parenchyma may be compromised unless compatible anatomical markers are included. It is also unclear if the tracer localization in PVS will be preserved after the extensive chemical treatment of the tissue employed in these techniques.

Other limitations are also noted. Firstly, periarterial spaces cannot be distinguished from perivenous spaces in the reconstructed 3D network which might be important to understand the relative importance of these pathways in brain solute/waste transport. In future studies, histological identification may be possible using fluorescent markers, e.g. Alexa Fluor 633^29^. MRI identification based on supra-paramagnetic contrast agents generated *in-situ* using reporter genes for cells lining the PVS is also a possibility^30,31^. Secondly, directionality of perivascular transport could not be inferred from the PVS network map. Tracer tracking by *in vivo* dynamic contrast enhanced MR imaging at ultra-high magnetic fields with high spatial and temporal resolution exploiting the sparseness in spatial-temporal domain could potentially resolve the transport directionality along PVS^32^. However. it should be noted that disruption of the dura mater, as well as infused flows have the potential to alter normal PVS flow rates and directions^33^. Future study of intraventricular transport dynamics could be assessed indirectly by sacrificing the animal at various time points post-infusion of the tracer. The advantage of the static time-point method for studying dynamics changes, compared to *in-vivo* dynamic contrast enhanced MRI studies, is that static time-point images can distinguish PVS transport from perfusion.

The current study focused on developing new protocols for visualizing PVS networks in normal rat brains. Developed methodologies can be used to measure variations in PVS networks with sleep and disease states which alter brain waste clearance. PVS network connectivity could be analyzed using methods developed for vascular networks to identify spatial maps of connectivity between different parts of the brain. PVS networks could also be used to better understand mechanisms driving transport by incorporating these structures into computational brain transport models that predict flow and mass transport along PVS pathways^34–36^. Overall this study is a significant advance that may be used to further explore perivascular transport at a mesoscopic scale.

## Materials and Methods

### PVS tracer

MR contrast agent gadolinium-diethylene-triamine pentaacetic acid labeled human serum albumin (Gd-albumin) was chosen to visualize the PVS network. Several properties of the contrast agent contribute to its utility as a PVS tracer. First, its large size reduces the diffusion rate into the adjacent parenchyma while the tracer is in the PVS, thereby increasing the MR sensitivity. The large size of the contrast agent also increases its r_1_ relaxivity which results in improved MR sensitivity^37^. Second, crosslinking of albumin protein to the extracellular matrix during the aldehyde fixation process preserves perivascular localization post-mortem. Third, the molecular weight of the tracer (MW~ 86 kDa) allows it to fully trace the glymphatic system. Solutes with molecular weight less than 100 kDa have been previously shown to leave the PVS into the parenchyma through the astrocytic endfeet clefts^38^. Gd-albumin for infusion was obtained from Robert Brasch’s laboratory in University of California, San Francisco (35 molecules of Gd-DTPA per albumin molecule)^37^. Infusate was prepared by diluting the 20 mM stock solution of Gd-albumin to 6 mM using 1x phosphate buffered saline (PBS), and for histology approximately 1 mg of Evans blue dye per 50 mg of Gd-albumin (~2 moles of Evans blue per mole of albumin) was added to the diluted solution. The mixing ratio was chosen to limit free Evans blue in the solution^39^, so that virtually all dye molecules are bound to Gd-albumin making them valid reporters for co-localized fluorescent dye distribution using confocal microscopy.

### Animal surgery

The tracer, Gd-albumin tagged with Evans blue dye (Gd-Alb-EB), was infused into the lateral ventricle of anesthetized rat brains to visualize PVS. Intraventricular tracer infusion is consistent with foundational perivascular transport studies by Rennels *et al*.^40^ and more recent studies by Muralidharan *et al*.^41^ It should be noted that our goal was not to maintain normal PVS flow patterns, rather infused tracers highlight connected PVS spaces.

Experiments were performed on 2-month old male Sprague-Dawley rats weighing 280–300 g (n = 7) using protocols and procedures approved by the University of Florida Institutional Animal Care and Use Committee. All methods were performed in accordance with relevant guidelines and regulations. Five rats were used for tracer infusion and two for naïve controls. Rats were anesthetized with 4% isoflurane in 1 L/min oxygen delivered with a nose cone, then the top of the head was shaved and disinfected with iodine and alcohol in preparation for surgery. The animals were then placed in a stereotaxic Kopf apparatus (Model 900 Small Animal Stereotaxic Instrument, David Kopf Instruments, Tujunga, CA, USA) with isoflurane adjusted between 1.5–3% in 1 L/min oxygen throughout the procedure to maintain anesthesia and normal breathing. A mid-sagittal skin incision was made to expose the bregma skull landmark, and a burr hole of 1.5 mm diameter was drilled above the lateral ventricle (AP: −0.72 mm, ML: +1.4 to +2.0 mm and DV: −3.8 mm from bregma) to insert the infusion needle.

A syringe pump (CMA Microdialysis AB, Torshamnsgatan, Kista, Sweden) with a 100 μL gas-tight syringe (Hamilton, Reno, NV, USA) attached to a 30-gauge needle (Hamilton, Reno, NV, USA) via PEEK tubing (ID = 65 μm, OD = 1.6 mm, length ~60 cm, Upchurch Scientific, Oak Harbor, WA, USA) was used to deliver 60 μL of the infusate at 1.5 μL/min. An RN-RN coupler (Hamilton, Reno, NV, USA) was placed between the PEEK tubing and the needle to monitor and remove bubbles in the infusion line. The infusion system was evaluated for accuracy before each surgery by measuring the mass of a target volume of infusate delivered at the experimental flow rate to account for losses and/or bubbles in the infusion system. The measured infusate mass corresponded to a less than 5% error from the target volume.

A large infusion volume (60 μL) was used to increase MR sensitivity. A corresponding flow rate of 1.5μL/min was chosen to reduce the infusion time required (40 minutes) and minimize diffusion of tracer into adjacent parenchyma. Previous studies in Sprague-Dawley rats that used a similar flow rate (1.6 μL/min) for similar duration (60 minutes) during intrathecal infusion of dextran showed minimal changes to the intracranial pressure^10^.

Following the end of infusion, the animal was immediately exsanguinated through transcardial injection using a 16-gauge blunt tip needle that introduced 100 mL of 0.9% sodium chloride solution followed by 300 mL of 4% formaldehyde PBS fixative solution. The rat carcass was post fixed at 4 °C for 2.5–3 days after which the brain was extracted and transferred to Fluorinert oil (FC-43, 3M Corp., St. Paul, MN, USA) in an 18 mm NMR tube (Wilmad-LabGlass, Vineland, NJ, USA) for MR imaging. Naïve control rats underwent the same procedure as described above except for stereotaxic surgery and tracer infusion. Commonly in conventional MRI studies conducted in aldehyde-fixed tissues^23,42^, tissue is rinsed in a PBS solution to minimize signal loss from T_2_ reduction due to the free fixative. Because of the limited fixation period used in this study, the tissue was not rinsed to prevent the loss of tracer not cross-linked with the extracellular matrix.

### MR imaging

Radio frequency (RF) signal at 750 MHz was transmitted and received using a 21 mm ID quadrature Litzcage probe^43^ (Doty Scientific, Columbia, SC, USA), and imaging was performed in a Bruker MRI system with 17.6 T vertical wide-bore magnet and Micro 2.5 gradient set connected to Avance III HD imaging console controlled by ParaVision 6.0 software (Bruker Biospin, Billerica, MA, USA). The presence of gadolinium tracer and sample quality were assessed using low resolution quantitative 2D multi-slice T_1_ and T_2_ measurements. Ten slices were acquired with 15 × 12 × 10 mm^3^ field of view (FOV), matrix size of 300 × 240 and 1 mm slice thickness to cover mid portion of the brain containing the ventricles. T_1_ was quantified using saturation-recovery spin echo sequence with TR = 5000, 3000, 2000, 1000, 500 and 250 ms, TE = 9 ms. T_2_ was quantified using multiple-spin-echo sequence with, TR = 5000 ms, TE = 10–120 ms in steps of 10 ms. Quantitative T_1_ scans were repeated at the end of an approximately 24-hour long high-resolution whole-brain PVS visualization scan described below to assess tracer diffusion during the imaging period.

Gadolinium uptake along perivascular spaces was visualized using 40 μm isotropic T_1_-weighted 3D spoiled and phase re-wound gradient echo dual-echo imaging sequence with TR = 100 ms, TE = 3/15 ms, flip angle = 50°, 20 × 16 × 12 mm^3^ FOV to cover the whole brain and cerebellum, matrix size of 500 × 400 × 300, and 7 averages. The PVS network was reconstructed from the shorter echo time image (i.e. TE = 3 ms) while the longer echo time image (i.e. TE = 15 ms) was used to verify susceptibility artifacts that may arise due to bubbles entrapped within the brain fissures during sample preparation and/or high concentration of the contrast agent which could appear hyperintense similar to that due to gadolinium. Spatial saturation bands were placed outside the FOV to prevent ghosting artifacts from water trapped in the gauze.

### 3D PVS reconstruction

Perivascular spaces were identified as bright regions in the whole-brain image data due to T_1_ shortening by the gadolinium tracer. Images were processed in the following order to reconstruct the perivascular network, (1) a combination of rodent brain extraction tool (rBET)^44^ in FSL software^45^ and ITK-SNAP software^46^ was used to remove gauze artifacts very close to the brain inside the FOV. (2) FMRIB’s linear image registration tool (FLIRT)^47,48^ in FSL software was used to register the data sets with Swanson’s rat brain atlas^49^. (3) Resulting stacks of whole-brain images were visualized in two-dimensions using maximum intensity projection (MIP) to reconstruct the perivascular network.

### Histology

After MR imaging, brain tissue was transferred to a solution of 30% sucrose in 0.1 M PBS for cryopreservation. Prior to tissue slicing, brightfield optical images of the brain were acquired using a dissection microscope (OPMI 1-FC, Carl Zeiss, Dublin, CA, USA) attached to a camera. Axial 250 μm thick frozen sections were then cut on a sliding microtome and placed on a microscope slide to image the distribution of Evans blue dye using confocal microscopy. Some slices were incubated in 1:1000 v/v DAPI (4′,6′-diamidino-2-phenylindole) in 1x PBS for 2–3 hours to stain cell nuclei for anatomical reference. Vectashield anti-fade aqueous mounting medium (Vector Laboratories Inc, Burlingame, CA, USA) was added to the wet slide topped with a #1.5 coverslip (Fisher Scientific, Hampton, NH, USA) to match the refractive index of the objective.

Laser scan confocal fluorescence images were acquired using a Nikon A1RMPsi-STORM 4.0 system (Nikon Instruments, Inc, Melville, NY, USA) with 10x and 25x objective lenses. Fluorophores were excited using 560.9 nm and 401.5 nm lasers with emission filters set at 695 ± 25 nm, 450 ± 25 nm to image Evans blue and DAPI distributions, respectively. Background autofluorescence images were acquired for anatomical structural reference with excitation at 488 nm and emission at 625 ± 25 nm. The images from multiple channels (Evans blue/DAPI or Evans blue/background) were then merged to form a whole-brain stacks of composite images which were projected using MIP.

### Statistical analysis

Statistical significance of the measured T_1_ at the infused lateral ventricle was evaluated using the 2-sided *t-*test with the hypothesis that the mean value is zero to account for noise/variability in the measurement. The analysis was run within the three treatment groups; 1) naïve controls, tracer infused rats 2) before and 3) after whole-brain MR imaging. Comparison was also made between the groups to assess the differences in T_1_ values at the infusion site.

## Supplementary information

Supplementary Information on Videos

Movie S1

Movie S2

Movie S3
