# Supplementary material for: MRI of Whole Rat Brain Perivascular Network Reveals Role for Ventricles in Brain Waste Clearance
Source: Sci Rep. 2019 Aug 7;9:11480. doi: 10.1038/s41598-019-44938-1 (PMC6685961; doi:10.1038/s41598-019-44938-1)
Supplement: Supplementary file 1 — Supplementary Information on Videos [file 41598_2019_44938_MOESM1_ESM.docx]

**MRI of Whole Rat Brain Perivascular Network Reveals Role for Ventricles in Brain Waste Clearance**

Kulam Najmudeen Magdoom^1^, Alec Brown^2^, Julian Rey^1^, Thomas H. Mareci^2^, Michael A. King^3,4^, and Malisa Sarntinoranont^1^

^1^Department of Mechanical and Aerospace Engineering, University of Florida, Gainesville, FL

^2^Department of Biochemistry and Molecular Biology, University of Florida, Gainesville, FL

^3^Department of Pharmacology and Therapeutics, University of Florida, Gainesville, FL

^4^Department of Veterans Affairs Medical Center, Gainesville, FL

# LIST OF SUPPLEMENTARY VIDEOS

Movie S1. 3D maximum intensity projection of the whole brain MR images of a naïve rat registered to the template rat brain atlas

Movie S2. 3D maximum intensity projection of the reconstructed whole brain perivascular network of a tracer infused rat registered to the template rat brain atlas

Movie S3. Coronal, sagittal, and horizontal maximum intensity projections of contiguous regions of interest (spanning 31 voxels in the projected direction) for the tracer infused brain perivascular network registered to the template rat brain atlas.
