# Supplementary figures and images for: MRI of Whole Rat Brain Perivascular Network Reveals Role for Ventricles in Brain Waste Clearance
Source: Sci Rep. 2019 Aug 7;9:11480. doi: 10.1038/s41598-019-44938-1 (PMC6685961; doi:10.1038/s41598-019-44938-1)

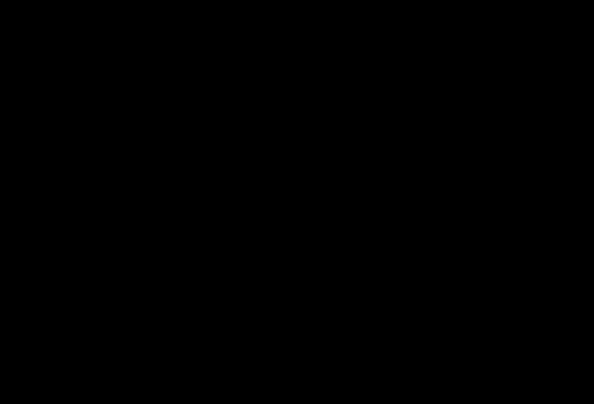

Supplement: Supplementary file 4 — Movie S3 [file 41598_2019_44938_MOESM4_ESM.gif]
